# Why and How the Old Neuroleptic Thioridazine Cures the XDR-TB Patient

**DOI:** 10.3390/ph5091021

**Published:** 2012-09-17

**Authors:** Leonard Amaral, Joseph Molnar

**Affiliations:** 1 Grupo de Micobacterias, Unidade de Microbacteriologia, Centro de Malaria e outras Doenças Tropicais (CMDT), Institute of Hygiene and Tropical Medicine, Universidade Nova de Lisboa, Rua Junqueira 100, 1349-008 Lisbon, Portugal; 2 Department of Medical Microbiology and Immunobiology, University of Szeged, Dóm tér 10, 6720 Szeged, Hungary; Email: molnar.jozsef@med.u-szeged.hu

**Keywords:** pulmonary tuberculosis, thioridazine, *in vitro* activity, *ex vivo* activity, cures the mouse of MDR-Mtb infections, XDR-TB, TDR-TB, cures the human of an XDR-TB infection, mechanisms of action, inhibitor of efflux pumps, enhances killing of intracellular Mtb

## Abstract

This mini-review provides the entire experimental history of the development of the old neuroleptic thioridazine (TZ) for therapy of antibiotic resistant pulmonary tuberculosis infections. TZ is effective when used in combination with antibiotics to which the initial *Mycobacterium tuberculosis* was resistant. Under proper cardiac evaluation procedures, the use of TZ is safe and does not produce known cardiopathy such as prolongation of QT interval. Because TZ is cheap, it should be considered for therapy of XDR and TDR-Mtb patients in economically disadvantaged countries.

## 1. Introduction

Tuberculosis is mainly an intracellular infection of the macrophage that is part of the alveolar unit of the lung. The infection is caused by *Mycobacterium tuberculosis* (Mtb) which is a steadfast human pathogen. As per the World Health Organisation (WHO), two billion of the World’s inhabitants are infected by Mtb [1]. However, active TB which is the progression of the intracellular infection to an extracellular status (organism breaks out of its intracellular prison) and is the infectious phase of the disease, takes place in roughly 5 to 10% of the globally infected. Unfortunately, the vast majority of active TB cases occur in India [2], China [3], and other low income areas of the globe; with some exceptions (for example Portugal [4] and Latvia [5]), active disease is far less common in Western Europe and the USA [6]. The frequency of progression of a TB infection to active TB status is markedly increased with co-infection with HIV or presentation of AIDS [7], and in the absence of major pathology, advanced age that is accompanied by a decrease of immunocompetence accounts for about 5% of all active TB. The mortality that TB exerts world-wide was in excess of 2 million for 2012 and is expected to rise. [1]. Nevertheless, given that epidemics of influenza kill as many as 10 million in a given year [8], TB is not a major killer and need not be a killer at all if the infection caused by antibiotic susceptible strains of Mtb is treated effectively with the two most effective antibiotics isoniazid (INH) and rifampicin (Rif) [9]. However, as elegantly pointed out by Zarir Udwadia [10], the development of multi-drug resistance (MDR) (resistance to INH and Rif) and its progression to more antibiotic resistant infections such as extensively drug resistant TB (XDR-TB) and now totally drug resistant TB (TDR-TB) is mainly due to incompetent therapy, but not all drug resistant infections are due to incompetent therapy, but rather to the very long period of therapy, often exceeding 15 continuous months and during this time, the normal rates of spontaneous mutations of targets of INH and Rif result in the accumulation of mutations [11] which render the infection immune to INH and Rif, and to other antibiotics as well as is the case for XDR-TB (resistance to INH, Rif, any fluoroquinolone and of the injectable TB drugs streptomycin, kanamycin and amikacin [12]. However, it must also be stated that not all drug resistance is due to mutations but rather to the over-expression of efflux pumps that extrude drugs before they reach their intended targets [13] or to the down-regulation of porins which limit the amount of drug that penetrates the cell envelope of the Mtb strain [14].

Therapy of antibiotic susceptible TB infections is effective when administered correctly, especially when it is associated with Directly Observed Treatments (DOTS) programmes [15]. However, therapy of the MDR-TB patient is problematic and extols high mortality, especially when the patient is co-infected with HIV or presents with AIDS [7]. Therapy of XDR-TB is very problematic with major mortality rates [16] and therapy TDR-TB, as defined by its name, almost always results in death [17].

At the present time, with the exception of adjunct use of the old neuroleptic thioridazine [18,19], there are no safe and effective drugs for therapy of the XDR and TDR-TB patient. It is the purpose of this mini-review to present the whole story of how thioridazine (TZ) was developed for the therapy of MDR-TB infections and why it is an effective drug for adjunct use with antibiotics to which the offending organism was initially resistant. Because of the dual mechanisms of action described in this mini review, any mutational response by the offending organism is irrelevant as would be the case for any drug that is to directly affect the survival of the organism. 

## 2. Phenothiazines

**Figure 1:**
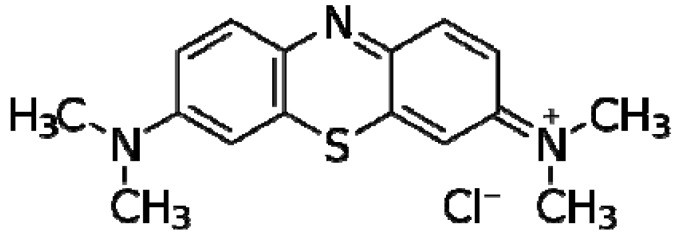
Structure of methylene blue (MB).

**Figure 2:**
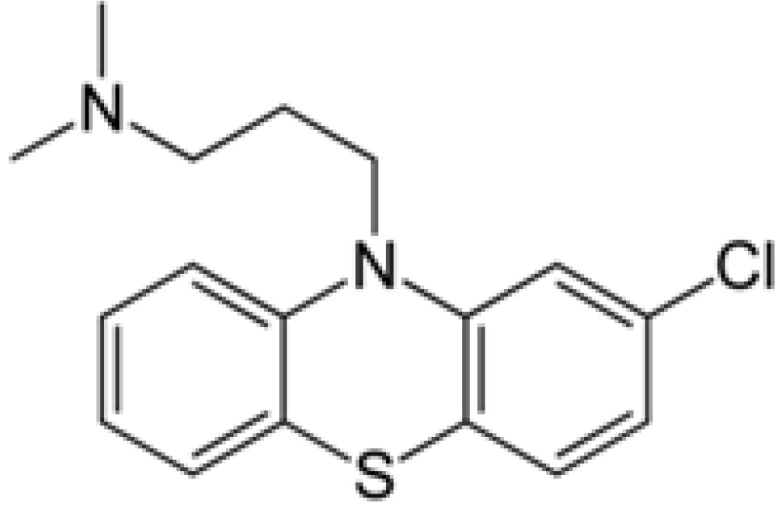
Structure of chlorpromazine (CPZ).

Phenothiazines are heterocyclic compounds whose structure is best exemplified by the dye methylene blue (MB, Figure 1). MB was studied intensively by the German physician-chemist Paul Erhlich in the 1890s and shown to have anti-malarial and antibacterial properties [20]. However, after the demonstration by Bodoni that when the dye was given to humans or other mammals, it would calm them [21], interest in the dye as a potential lead compound for the synthesis of a true neuroleptic took precedence over its antimicrobial properties. Nevertheless, it took half a century for the synthesis of the first commercially available colourless neuroleptic chlorpromazine (CPZ, Figure 2) by the French chemist Charpentier [22] and soon after its release as Largactil by Rhone Poulenc Inc. in the middle 1950s, it was used world-wide for the control of psychosis. As a consequence of this extensive use, the activity of CPZ against a wide gamut of microorganisms was studied [23]. Moreover, because of the plethora of negative side effects it produced, the study of the biological effects CPZ has generated almost 20,000 published studies listed by PubMed, second only to aspirin (over 50,000). Among the important biological properties of CPZ are its *in vitro* and *in vivo* inhibition of intracellular pathogens such as leishmania [24,25], trypanosomes [26,27], amoebae [28,29,30,31], *in vitro* inhibition of cancer cells [32,33], induces of apoptosis of cancer cells [34,35], inhibits efflux pumps of multi-drug resistant bacteria [36,37], causes the elimination of plasmids that carry antibiotic resistant genes from important pathogenic bacteria [38,39,40], inhibits enzymes when studied [41], and many other cellular activities that are too numerous to mention. Nevertheless, as recently reviewed [23], the activities noted for CPZ lie in the side chains of the molecule [23] and have guided the evolution of this phenothiazine as an antimalarial agent [42].

The activities of CPZ, relevant to the theme of this mini review, lie in their anti-microbial properties and these are discussed and traced in the development of thioridazine as an anti-tubercular drug in the sections that follow.

## 3. Anti-Tubercular Activity of CPZ and Other Phenothiazines

Within a year after the introduction of CPZ for therapy of psychosis, CPZ [43] and other phenothiazine derivatives [44] were observed to improve therapy of pulmonary tuberculosis. These reports were followed with many demonstrations that indeed CPZ could be used as an adjunct for rapidly curing the TB patient [45,46,47,48]. However, these studies took place at the time that INH and rifampicin had been introduced as effective therapeutic agents for management of tuberculosis. Moreover, the side effects from CPZ were numerous and significant [49], hence, why use it if other less noxious compounds were presently available. Nevertheless, interest in CPZ as an anti-tubercular agent remained as evident from *in vitro* studies conducted during the next three decades [50]. However, the concentrations that produced *in vitro* inhibition of replication of Mtb were extremely high (ranged from 15 to 25 mg/L) [51,52,53] and well beyond the maximum safe serum level (0.5 mg/L) achieved with chronic therapy of the psychotic patient. The demonstration that a concentration of CPZ in the medium that was within clinical reach could effectively kill intracellular Mtb by non killing human macrophages [54], sparked interest in the potential that CPZ offered for therapy of tuberculosis; especially multi-drug resistant infections. Moreover it explained why therapeutic doses of CPZ could effectively cure tuberculosis infections noted during the first decade of CPZ use world-wide. 

**Figure 3:**
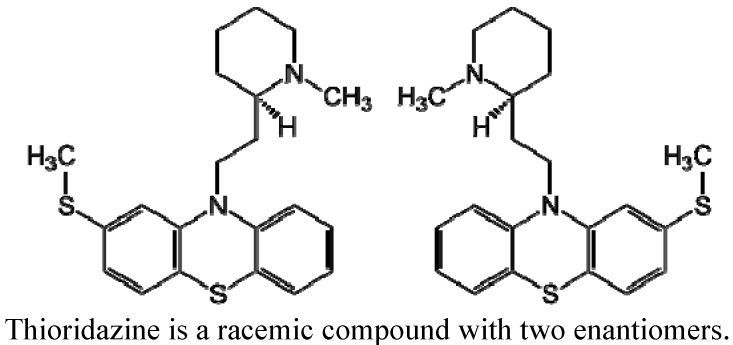
Structure of thioridazine (TZ).

During the 1980s New York City experienced a quadrupling of the new cases of active TB infections with more than half of these presenting with an MDR phenotype [55]. The need for effective anti-tubercular drugs was urgent, but none were in the pipeline due to little interest of pharmaceutical companies. Moreover, the problems that CPZ posed were still insurmountable. However, because thioridazine (TZ, Figure 3) is an effective neuroleptic with fewer significant negative side effects, the *in vitro* activity of TZ against a panel of Mtb strains that were resistant to as many as five antibiotics was examined and the phenothiazine was shown to be as effective in the inhibition of replication of Mtb as CPZ [56]. Nevertheless, the concentration of TZ needed to inhibit replication was of the order of 30 mg/L and therefore, clinically irrelevant. The demonstration that a concentration of TZ in the medium (0.1 mg/L) which was lower than that used for chronic therapy of the psychotic patient caused the killing of intracellular Mtb by non-killing macrophages [57], was soon followed by studies that showed that TZ could cure the mouse infected with antibiotic susceptible Mtb [58] as well as by an MDR Mtb strain [59]. The latter study also showed, as was previously demonstrated *in vitro* [60], that TZ could enhance the *in vivo* effectiveness of INH and Rif when used as therapy of the MDR Mtb infected mouse [59]. Proof in support that TZ cures extensively resistant TB infections was recently presented [61]. This latter retrospective study was performed on 17 non-AIDS pulmonary adult patients with XDR-TB admitted to a referral treatment centre for infectious diseases in Buenos Aires, Argentina from 2,002 through 2,008. A combination of linezolid, moxifloxacin and thioridazine was applied in the treatment of 12 patients. Thioridazine was initially administered at a daily dose of 25 mg for two weeks, thereafter the dose was increased to 25 mg weekly until it reached 200 mg/day under strict cardiac monitoring in order to survey for eventual cardiac adverse events. Eleven patients met the recovery criteria with more than two years of follow-up after treatment completion. Thioridazine was discontinued in one patient with pancytopenia and in another with allergic dermatitis. Although cardiac adverse effects have been reported previously, no prolongation of QT interval or any other heart complication was observed. Another recent study showed that employing a similar dose schedule, but limiting the final dose to 75 mg/day, improved the quality of life of XDR TB patients, *i.e.*,patients regained their appetite, put on weight, night sweats were reduced or obviated, the anxiety produced by the infection was obviated, *etc*. [62] and these authors have therefore recommended that TZ be considered as a salvage drug for therapy of antibiotic non-responsive XDR TB patients whose prognosis is serious [62]. This study, as was the case with the Abbate *et al.* study [61] also showed that none of the patients presented with any evidence of prolonged QTc intervals or with any other cardiopathy [62]. Clearly, TZ merits serious consideration as an adjunct for therapy of pulmonary TB infections that do not respond to available drugs.

## 4. How Does TZ Cure Drug Resistant Pulmonary Infections Such As XDR-TB and Probably TDR-TB?

### 4.1. The Role of TZ as an Inhibitor of Mtb Efflux Pumps

Multi-drug resistance of Mtb can be due to accumulated mutations of antibiotic targets, to down-regulation of porins [14] and to over-expression of efflux pumps that excrete two or more unrelated classes of antibiotics [13]. Although the degree that efflux pumps contribute to the multi-drug phenotype of MDR, XDR and TDR Mtb strains is not known, if we can extrapolate from other studies, it is certain that a major fraction of multi-drug resistance is due the over-expression of its efflux pumps [13,14,63,64]. Among the efflux pump genes that respond to INH exposure by over-expressing their total mRNA, are *mmpL7*, *p55*, *efpA*, *mmr*, *Rv1258c* and *Rv2459* [13]. The activity of each of these genes is reduced by exposure to TZ [13]. TZ also inhibits the products of each of these genes, and these inhibitions render the MDR Mtb strain susceptible to antibiotics to which it was initially resistant [13,14,63,64]. Moreover, TZ has been shown to inhibit the expression of many essential genes of Mtb [65,66] and can also kill dormant Mtb [67,68]. Although not yet proven, it is quite certain that since TZ is concentrated by lysosomes [69,70,71], the *in vivo* activity of TZ that leads, at least in part, to the cure of the Mtb infected mouse [58,59], must be due to the concentrated effect of the compound within the phagolysosome that has entrapped the bacterium, as previously supported by prior studies [71]. Moreover, the activity of thioridazine has also been shown to enhance the killing of intracellular antibiotic susceptible and MDR-Mtb [57] and XDR-Mtb [72,73,74,75,76,77] by non-killing human macrophages.

### 4.2. The Mechanism by Which TZ Enhances the Killing of Intracellular Mtb

The pulmonary macrophage, unlike other macrophages such as the neutrophil, has little killing activity of its own. Consequently, the entrapped Mtb within the phagolysosome vacuole remains viable for many decades. The mechanism by which TZ enhances killing of intracellular Mtb has been postulated to be due to the TZ inhibition of potassium ions from the phagolysosomal vacuole [72,73,74,75,76]. The retention of potassium ions promotes the acidification of the phagolysosomal vacuole which in turn activates the inert hydrolases resulting in the degradation of the entrapped Mtb organism [72,73,74,75,76]. This enhanced killing by non-killing macrophages results in a totally new concept for the therapy of pulmonary TB as well as other intracellular infections mentioned in this review. This new concept rather than targeting the intracellular organism, targets the inert hydrolytic system of the macrophage. Consequently, this effect by-passes any mutational response of the entrapped Mtb as would be the case for other drugs that target the organism itself.

## 5. Conclusions

TZ has been shown and confirmed to have *in vitro*, *ex vivo* and *in vivo* activity against all encountered strains of Mtb. TZ has been shown to cure the XDR-TB patient when used in combination with antibiotics to which the strain was initially resistant. TZ has also been shown to vastly improve the quality of life of the XDR-TB patient. The application of TZ for therapy of the XDR TB patient when proper evaluation of cardiac function is undertaken, produces no cardiopathy. TZ is cheap and certainly affordable by low income countries. Consequently, TZ is recommended for therapy of antibiotic unresponsive XDR-TB patients. Moreover, because of the dual mechanism of action, TZ is expected to produce similar cures in the TDR-TB patient. For countries such as India, it must be considered now. Nevertheless, because the concentration of thioridazine needed to inhibit the extracellular *Mycobacterium tuberculosis* infection exceeds the safe limits of its clinical use, so it cannot be used to treat a tuberculosis infection that is extracellular or in the process of dissemination to other sites of the body.
